# A combined machine learning and finite element modelling tool for the surgical planning of craniosynostosis correction

**DOI:** 10.1371/journal.pone.0336473

**Published:** 2025-12-05

**Authors:** Itxasne Antúnez Sáenz, Ane Alberdi Aramendi, David Dunaway, Juling Ong, Lara Deliège, Amparo Sáenz, Anita Ahmadi Birjandi, Noor UI Owase Jeelani, Silvia Schievano, Alessandro Borghi

**Affiliations:** 1 Great Ormond Street Institute of Child Health, London, United Kingdom; 2 Biomedical Engineering Department, Mondragon Unibertsitatea, Loramendi Kalea, Arrasate, Spain; 3 Great Ormond Street Hospital, London, United Kingdom; 4 Department of Engineering, Durham University, Durham, United Kingdom; University of Vermont College of Medicine, UNITED STATES OF AMERICA

## Abstract

Craniosynostosis is a medical condition that affects the growth of babies’ heads, caused by an early fusion of cranial sutures. In recent decades, surgical treatments for craniosynostosis have significantly improved, leading to reduced invasiveness, faster recovery, and less blood loss. At Great Ormond Street Hospital (GOSH), the main surgical treatment for patients diagnosed with sagittal craniosynostosis (SC) is spring assisted cranioplasty (SAC). This procedure involves a $15\times 15{\text{mm}}^{2}$ osteotomy, where two springs are inserted to induce distraction. Despite the numerous advantages of this surgical technique for patients, the outcome remains unpredictable due to the lack of efficient preoperative planning tools. The surgeon’s experience and the baby’s age are currently relied upon to determine the osteotomy location and spring selection. Previous tools for predicting the surgical outcome of SC relied on finite element modeling (FEM), which involved computed tomography (CT) imaging and required engineering expertise and lengthy calculations. The main goal of this research is to develop a real-time prediction tool for the surgical outcome of patients, eliminating the need for CT scans to minimise radiation exposure during preoperative planning. The proposed methodology involves creating personalised synthetic skulls based on three-dimensional (3D) photographs, incorporating population average values of suture location, skull thickness, and soft tissue properties. A machine learning (ML) surrogate model is employed to achieve the desired surgical outcome. The resulting multi-output support vector regressor model achieves a ${\text{R}}^{2}$ metric of 0.95 and MSE and MAE below 0.13. Furthermore, in the future, this model could not only simulate various surgical scenarios but also provide optimal parameters for achieving a maximum cranial index (CI).

## Introduction

Craniosynostosis is a condition that affects approximately 1 in 2000 babies in the UK [1]. Babies with craniosynostosis not only have an abnormal head shape but may also experience impaired brain growth. In most cases, surgical intervention is required to correct the shape and facilitate proper growth [2].

Craniosynostosis can be classified into two groups: syndromic craniosynostosis (associated with a genetic abnormality), and non-syndromic craniosynostosis which represents 92.21% of all cases [3].

Craniosynostosis can also be classified according to the affected suture. The most frequent one is sagittal craniosynostosis (with a male-to-female ratio of 3.1 to 1 [2]) where the head midline suture (sagittal suture) is involved. The early fusion of the sagittal suture produces a long and narrow head(scaphocephaly) [2]. In this study, we use the term sagittal craniosynostosis to describe the pathological ossification of the sagittal suture, and scaphocephaly to describe the resulting cranial morphology.

The diagnosis is commonly done by means of a radiologic examination. Following primary care physician diagnosis, a plain skull radiographic series is ordered to confirm the sutural fusion. Afterwards, computed tomography (CT) imaging is recommended to obtain more information [4]. Nevertheless, it has been demonstrated that 3D-CT images are better than plain radiographs or standard CT [4]. The alternatives for avoiding radiation are detecting the suture fusion employing ultrasound technology, whose reliability is still debated [4], or the physical examination (accurate in 98% of the cases).

Three-dimensional (3D) photography is a non-invasive and non-ionising method used as an alternative to other 3D imaging types to acquire surface information of the human body [5,6]. This technology has been successfully employed in cranial and maxillofacial surgery to compute and quantify changes after surgeries [5,7].

**Fig 1:**
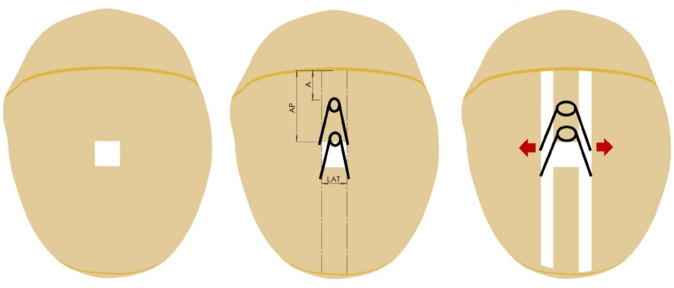
Sketch of the SAC procedure.

There are several ways in which craniosynostosis can be treated. Invasive techniques include total calvarial remodelling (TCR) and distraction for fronto-orbital or posterior cranial surgery. Both techniques consist on trimming and cutting different parts of the skull to form normal shapes. Nevertheless, also non-invasive techniques are available for treatment. Non-invasive techniques include the Endoscopic strip craniectomy followed by helmet therapy which consists of making small incisions to remove the fused suture. The patient must wear a helmet during the following months after surgery to continue with the modelling. Another non-invasive technique, that currently is the most used one to treat SC at GOSH is spring assisted cranioplasty (SAC). The procedure starts with an incision between 80 and 100 mm over the top of the head that will expose the skull. Once the skull is exposed, an osteotomy will be performed to remove a portion of the fused sagittal sutures and the springs distractors placed as displayed in Fig 1.

**Fig 2:**
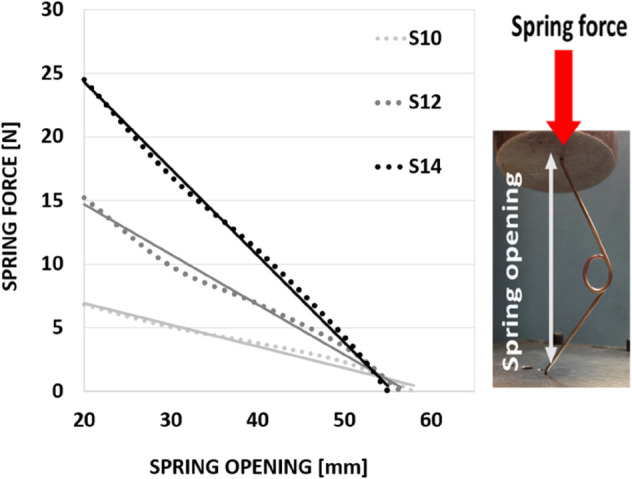
Springs Force vs Opening curve followed by the springs in the uncrimping phase.

The springs used at GOSH to correct SC, are stainless steel wires with a central loop and an initial opening of 60 mm [8,9]. The springs are crimped before insertion and then they passively expand. During the uncrimping phase the springs follow a Hookean behaviour exerting an outward force directly proportional to the amount of compression they have undergone as displayed in Fig 2.

In previous surgical planning processes, finite element modelling (FEM) has been used to obtain reasonably accurate results. However, this approach requires the use of CT imaging, along with engineering expertise and lengthy calculation times [9–11]. Therefore, to date the surgeon selects the surgical parameters, spring model, and location in the operating theatre based on their own experience and the age of the baby resulting in unpredictable outcomes [9]. Thus, a more efficient tool is required to select the appropriate surgical parameters, spring model and location to achieve the best possible result.

Recently, AI-FEM surrogate models (based on machine learning (ML) and Deep Learning (DL)) have been developed for preoperative planning of surgical procedures - such as in the cardiovascular field [12]. Surrogate models are hybrid models trained using a wide range of FEM simulations, enabling real-time predictions of stresses and deformations without extensive calculations [13].

Statistical shape modelling (SSM) is a dimensionality reduction technique used to describe anatomical variations within a subject population. It has been used in the past for organ, tumour or bone visualization, surgical planning or quantification of disease progression [14]. SSM is based on the quantification of the shape variation after Principal Component Analysis (PCA) is applied in a set of shape vectors [15]. PCA will determine the main components to describe the shape variation within the population. The components that describe the shape variation of the first set of samples can be optimised to fit new individuals, creating a familiar active shape model (ASM) [15]. This method has already been applied in craniofacial surgery [16,17].

Considering the favourable outcomes achieved by FEM in predicting the surgical outcome of craniosynostosis, we hypothesise that an AI-FEM surrogate model could be potentially employed to maintain the positive results offered by FEM for this application, while eliminating the associated challenges, namely CT imaging, engineering expertise, and time consumption. While ML surrogate models have been explored in other biomedical fields, to our knowledge, their direct application to (SAC) has not been reported. AI has previously been applied in craniofacial surgery for preoperative planning [18], but this is the first time surrogate models are being investigated in this context. By introducing an AI-FEM surrogate tailored to SAC, this work represents not only a methodological advance but also a clinical innovation with the potential to transform how surgeons select springs and develop the preoperative planning.

## Materials and methods

### Data collection

**Fig 3:**
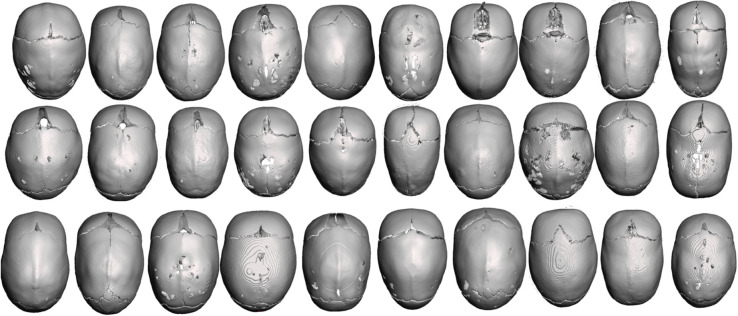
Upper view of the CT scans of the population.

**Fig 4:**
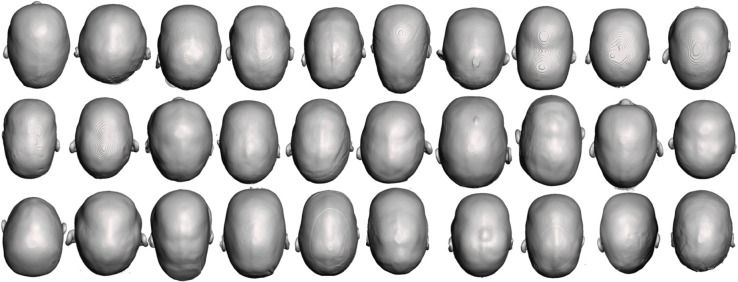
Upper view of the 3D scans of the population.

In this retrospective study, CT scans were processed to extract 3D skull shapes (Fig 3) and 3D head shapes (Fig 4) of 30 non-syndromic sagittal craniosynostosis patients aged 5.8 ± 1.15 months at the time of surgery which were intervened at GOSH between December 2011 and June 2022 were employed. Written parental consent was obtained for the participation in this retrospective study, and all data was previously anonymised.Ethical approval was obtained for the use of patient image data for research purposes (UK REC 15/LO/0386—Research Ethics Committee approval—Study No. 14DS25). The data was accessed between November 2022 and July 2023.

The CT images provided valuable population data on suture location and positioning, as well as skull and soft-tissue thickness.

**Fig 5:**
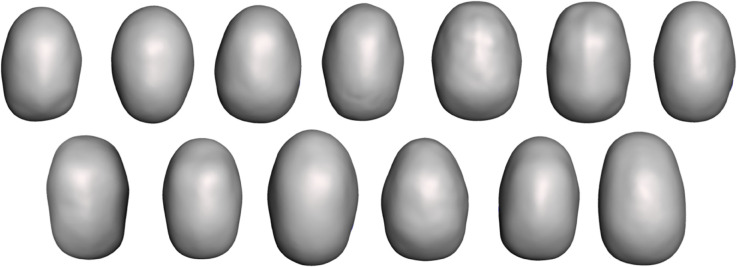
Upper view of the 3D scans of the validation population.

Additionally, a second separate population (Fig 5) consisting of 13 non-syndromic sagittal craniosynostosis patients aged 5.1 ± 1.0 with the preoperative and follow-up (3 weeks after surgery) 3D photographs operated at GOSH with SAC procedure between 2015 and 2017 was used for validation.

### Image processing

#### CT image processing.

Head and skull models were extracted in STL format (standard triangulation language), with a 70% reduction in the number of triangles to mitigate computational costs in subsequent stages. Artifacts in CT images, stemming from both sensor limitations and areas devoid of bone, are common and were covered using Autodesk MeshMixer.

Each skull model was cut along the nasion-upper auditory meatus plane, which effectively delineates the neurocranium from the viscerocranium. Anatomical measurements were taken, including the length of the skull, distance between sutures, and angles (following the protocol described in [11]). All the distances were converted to ratios to ensure robustness. Each model was split into outer and inner skull to determine individual skull thickness (for this purpose, the lower 25% was excluded to mitigate potential errors introduced by spurious anatomical structures). Average population skull thickness (*t*_*skull*_) was calculated.

Each head model was processed similarly: triangle reduction was followed by artifact reduction (performed in Autodesk Meshmixer). Each model was cut along the nasion-tragion plane. Soft tissue thickness was computed using surface distance between outer skull and head model for each patient, following the protocol described in [11]. Average population skin thickness (*t*_*skin*_) was calculated.

#### 3D Image processing.

Each processed head model was imported into Solidworks ^©^ where a Non-Uniform rational *β*-Spline (NURBS) was generated ; subsequently, it was uniformly internally offset by *t*_*skin*_ (i.e. the population mean of soft tissue thickness). This process yielded a synthetic skull. Sutures were artificially created on the model following the method by [11], assuming a width of 2 mm [19]. The final synthetic skull model was composed of frontal bone, parietal bone, occipital bone, coronal and lambdoid suture as displayed in Fig 1.

Ultimately, the osteotomy was replicated on the parietal bone, following the same method previously published by our group [10,11]. Briefly, a central point was established at the center of the coronal suture, serving as the origin for all measurements. Subsequently, a rectangle (spanning from the coronal suture to the lambdoid suture) was outlined and cut out of the parietal bone, including four notches (having a 5 mm diameter) replicating the surgical point of insertion of the cranioplasty springs [20].

Osteotomies were defined using three measurements: First, the distance between the reference point and the closest notches (anterior spring location - *A*), the distance between the reference point and the other notches (*AP*) and finally the width of the rectangle (*LAT*) as displayed on Fig 1.

### Finite element modelling

The assemblies were imported into Ansys Benchmark ^©^. Edges where forces and constraints were applied had to be selected and grouped using named selections. This selection encompassed the fixed bottom edges, the four individual notches where the springs were inserted, and the borders of the five distinct parts that were bonded together.

The material properties assigned to each part were defined based on [21]. Frontal, parietal and occipital bones were assigned isotropic elastic material properties (Young Modulus and Poisson’s ratio) and viscoelastic properties (described through a Prony series).

The spring action was replicated using linear spring conditions, described in terms of stiffness and free length, with both parameters being parametrised to facilitate adjustments during the design of experiments. The mechanical properties of the three different spring models were obtained from [9].

To prevent unrealistic behaviour, bond restrictions were enforced between all parts. In terms of meshing, a standard size of 1.5 mm was applied to bone, and 1 mm to sutures. The preferred mesh method is triangulation, and all parts have a consistent width of *t*_*skull*_ mm.

The design of experiments (DoE) was used to generate different surgical configurations and hence increase the dimensionality and variability of the population. The scenarios were automatically created using the optimal-space-filling configuration of Ansys Benchmark ^©^.

During the DoE stage, constrains were applied to each parameter. For springs, the range was determined based on the real springs range, while osteotomy parameters were limited according to the specifications outlined in Table 1.

**Table 1:** Parameters and ranges.

Parameter	Value (%)
**A**	[18-30]
**AP**	[47-63]
**LAT**	[10-25]

The DoE assigned different values for the different parameters. Surgical parameters followed a normal distribution and the springs models a uniform one. After configuring all parameters, DoE was conducted for each of the 30 patients, encompassing 80 ± 5 distinct surgical configurations for each. This yielded a total of 2356 FEM outputs. All of these outputs were automatically exported in Compact Database (cdb) format.

### Data acquisition

The data mining process started with the conversion of the cdb files into actual meshes. This process was automated, involving the identification of node and element locations within these files. Then, meshes underwent an external offset of *t*_*skin*_ to recover the soft-tissue shape and were saved as STLs.

The osteotomy gap was closed using Materialise 3-Matic ^©^, which maintained the natural roundness of the skull. To achieve point to point correspondence, a template was fitted to each output mesh using Non-rigid Iterative Closest Point (NRICP) algorithm. This template standardizes the number of nodes and elements constituting each of the skulls, which was crucial for the correct implementation of Statistical Shape Modeling (SSM).

#### Statistical Shape Modeling (SSM).

SSM is a technique to describe shapes based on a mean shape $\overset{―}{M}$ with a set of modes of variation. These variations are represented by the eigenvectors $\Phi =[\varphi_{1},\varphi_{2},...,\varphi_{k}]$, where each $\varphi_{i}$ corresponds to a principal component (PC) (mode of shape variation). The coefficients $b=[b_{1},b_{2},...,b_{k}{]}^{T}$ describe the contribution of each mode. A new shape instance ***M***_***p***_ can then be expressed as in Eq 1 [22].

$$M_{p}=\overset{―}{M}+\Phi b$$ (1)

There are 30 shapes corresponding to pre-operatory stage and 2356 from simulated surgical outcomes. The SSM was separately conducted on these two groups with *N*_*preop*_ = 30 and *N*_*postop*_ = 2356. Following the procedure described in [22] the two mean shapes of input and outputs sets were achieved $\overset{―}{M}$ $\in {\mathbb{R}}^{N\times 3}$.

The variability was computed for each of the sets by rearranging the nodes and elements of each i-th shape into column vectors.


$${\text{M}}_{\text{preop}}\in {\mathbb{R}}^{N_{preop}\times 3}$$

$${\text{M}}_{\text{postop}}\in {\mathbb{R}}^{N_{postop}\times 3}$$

Leading to the following expressions for preoperative shapes:


$$m_{preop,i}\in {\mathbb{R}}^{N_{preop}\times 1}$$

mpreop∈ℝNpreop×1

And postoperative shapes:


$$m_{postop,i}\in {\mathbb{R}}^{N_{postop}\times 1}$$

mpostop∈ℝNpostop×1

After rearranging the following two expressions are obtained:

$${\text{m}}_{preop,i}=[x_{1},y_{1},z_{1},x_{2},y_{2},z_{2},...,x_{N_{preop}},y_{N_{preop}},z_{N_{preop}}{]}^{T}$$ (2)

$${\text{m}}_{postop,i}=[x_{1},y_{1},z_{1},x_{2},y_{2},z_{2},...,x_{N_{postop}},y_{N_{postop}},z_{N_{postop}}{]}^{T}$$ (3)

The new representation of the shapes set was computed by the deviation between the individual shapes and the corresponding mean as follows:

mpreop,d=mpreop,i−mpreop (4)

mpostop,d=mpostop,i−mpostop (5)

These vectors were rearranged again into two matrices:


$${\text{M}}_{\text{preop,d}}=[m_{preop,1},m_{preop,2},...,m_{preop,N_{preop}}]$$

$${\text{M}}_{\text{postop,d}}=[m_{postop,1},m_{postop,2},...,m_{postop,N_{postop}}]$$

Eigenvectors ($\varphi_{i}$) and eigenvalues ($\lambda_{i}$) were computed from the covariance matrices of the ***M***_***preop*,*d***_ and ***M***_***postop*,*d***_. The eigenvectors are given by:


$$\varphi_{i}\in {\mathbb{R}}^{N_{preop}\times 1}i\in [1,N_{preop}-1]$$

$$\varphi_{i}\in {\mathbb{R}}^{N_{postop}\times 1}i\in [1,N_{postop}-1]$$

The eigenvalues quantify the variance explained by their corresponding eigenvectors. The variances are sorted based on the descriptive variance and corresponding PCs rearranged. The proportion of variance explained by the *i*-th eigenvalue is:

$$\lambda_{explained,i}=\frac{\lambda_{i}}{\sum_{i=1}^{N-1}\lambda_{i}}$$ (6)

Finally, SSM was conducted separately on the 30 input shapes and on the 2356 outcome shapes, which characterized the shapes in terms of a mean shape and variations. To efficiently determine the number of modes required to describe a population, a cumulative distribution function (CDF) was plotted. The modes describing 94% percentage of variation were selected.

All the collected data was then compiled in a single dataset to train the algorithms. The dataset included the patient’s age at surgery time expressed in days, surgical parameters (in ratios), springs’ stiffness and free length, and the modes describing both input and output shapes. This compilation resulted in a dataset of size 2356x30.

### Machine learning

The dataset was split into training and testing sets with a test size of 0.33. Seven multi-output machine learning algorithms were evaluated with the main objective of predicting output shapes based on input variables, including age, surgery time, surgical parameters, spring characteristics, and input modes. The tested models included Linear Regression (LR) (used as the baseline), Decision Tree (DT), Random Forest (RF), XGBoost (XGB), Support Vector Machine (SVM), Gradient Boosting (GB), and AdaBoost (AB).

**Fig 6:**
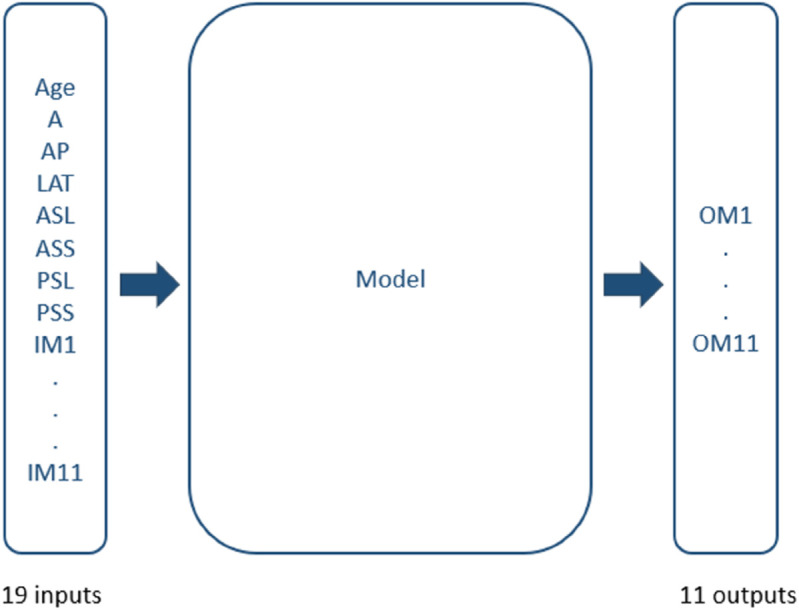
Multi-output regression sketch. Multi-output regression sketch.

Since some of these models rely on distance-based calculations, all variables needed to be on the same scale. Therefore, a standard scaler was applied to normalize the input data. The structure of the multi-output regression models is illustrated in Fig 6.

Model performance was evaluated using *R*^2^, MAE and MSE metrics, along with a 5-fold cross-validation approach. Models exhibiting high variability during 5-fold cross-validation were discarded, as well as those with a lower *R*^2^ score than the baseline LR model. Finally, hyperparameter tuning was performed on the four best-performing models using Bayesian search, and their accuracy and error metrics were assessed using the test set. The Bayesian search was done in 5 seeded folds with 50 iterations for the parameters shown in Table 2 and no priors were used.

**Table 2:** Parameters and distributions used during the Bayesian search optimisation.

Model	Parameter	Distribution	Value range	Optimal Value
**RF**	n_estimators	Uniform	[10-150]	103
	max_depth	Uniform	[5-20]	17
	min_samples_split	Uniform	[2-10]	2
	min_samples_leaf	Uniform	[1-5]	1
**XGB**	booster	Categorical	gbtree, gblinear, dart	dart
	eta	Uniform	[0.01-1]	0.18
	gamma	Uniform	[0-0.6]	0.36
	max_depth	Uniform	[1-100]	80
	sampling_method	Categorical	uniform, subsample, gradient_based	uniform
**GB**	n_estimators	Uniform	[10-500]	500
	learning_rate	Uniform	[0.01-1]	0.12
	loss	Categorical	squared_error, absolute_error, huber, quantile	huber
	criterion	Categorical	friedman_mse, squared_error	squared_error
**SVM**	degree	Uniform	[1-9]	1
	gamma	Categorical	scale, auto	auto
	epsilon	Uniform	[0-5]	0
	kernel	Categorical	linear, poly, rbf, sigmoid	rbf
	C	Uniform	[0.01-5]	1.85

#### Validation using real data.

The validation population was used for the final validation of the selected model. The postoperative 3D photographs were scaled down to omit the growth factor that was not considered at the time of simulation.

## Results

### Image processing

The skull thickness *t*_*skull*_ and the skin thickness *t*_*skin*_ of the population were 2.02±0.33 mm and 3.42±0.51 mm respectively.

### SSM

**Fig 7:**
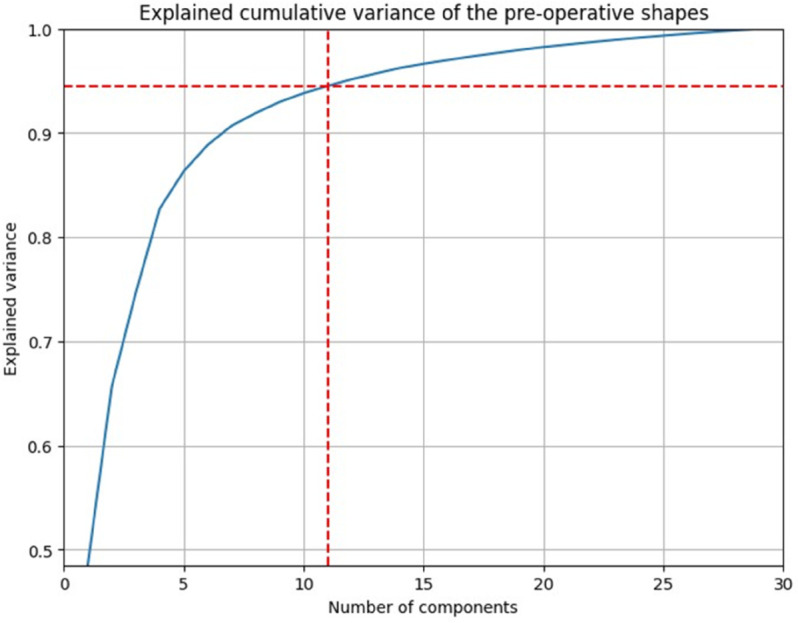
CDF of input modes.

**Fig 8:**
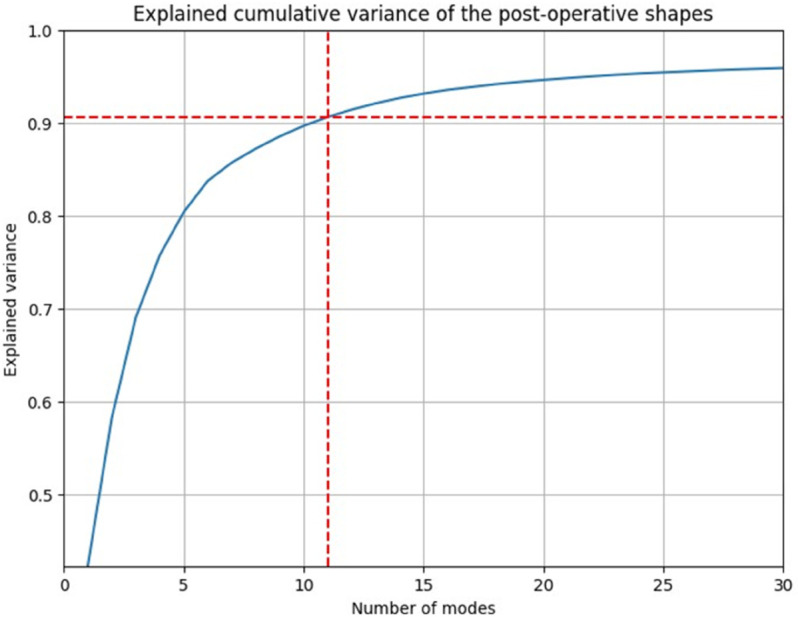
CDF of output modes.

For the input shapes, 11 modes were chosen to comprehensively describe the shapes, accounting for 94% of the variation. Similarly, the same number of modes (11) were selected for the output, but in this case, they accounted for 90% of the shape variation as it is illustrated in Figs 7 and 8.

**Fig 9:**
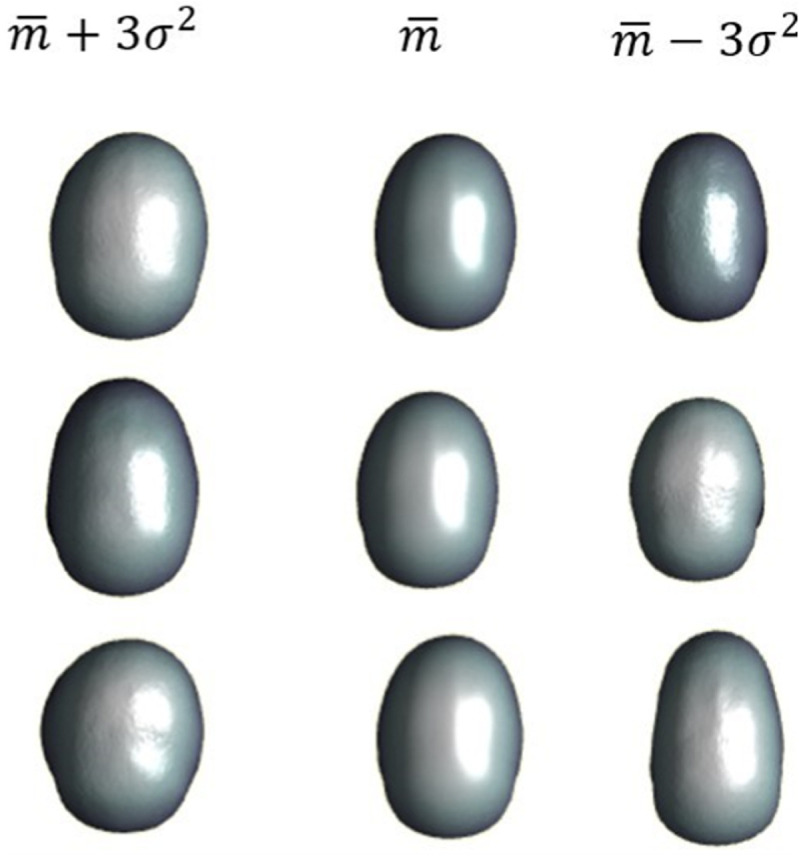
Influence of the input modes.

**Fig 10:**
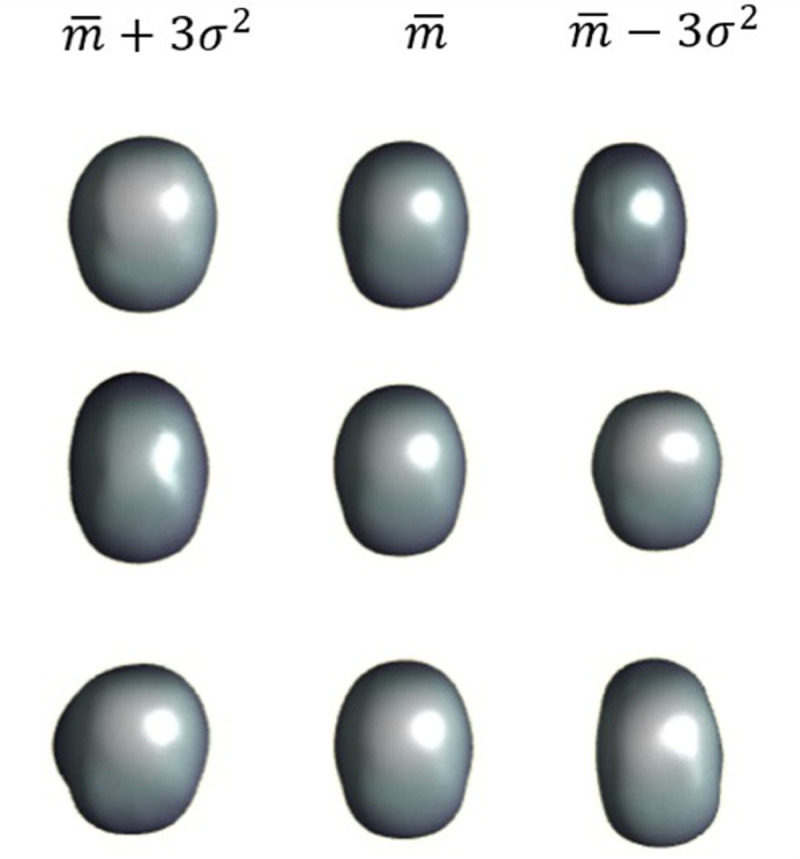
Influence of the output modes.

In the Figs 9 and 10 the influence of the first 3 modes for input and output can be observed.

### Predictive model

**Fig 11:**
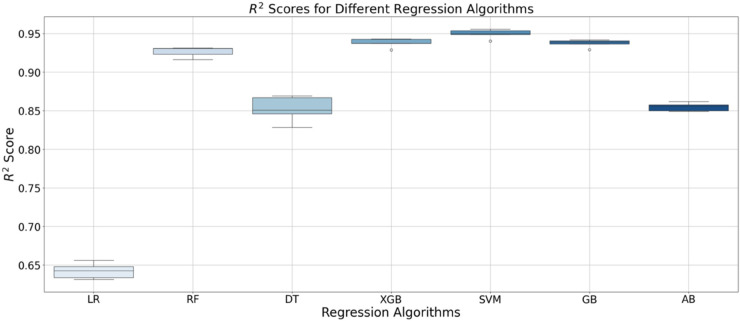
${\text{R}}^{2}$ values scored by the different regression algorithms. Linear Regression (LR), Random Forest (RF), Decision Tree (DT), X Gradient Boosting(XGB), Support Vector Machine (SVM), Gradient Boosting (GB) and AdaBoost (AB).

In the baseline shown in Fig 11, the variation of the ${\text{R}}^{2}$ value across the 5-folds for different algorithms can be observed. DT exhibited the highest variance, and, taking LR as the baseline, all the remaining models achieved accuracies more than 20 percentage points higher.

The Table 2 summarizes the search space used for Bayesian hyperparameter optimization for the remaining algorithms, along with the final selected optimal hyperparameter values and their distributions.

**Fig 12:**
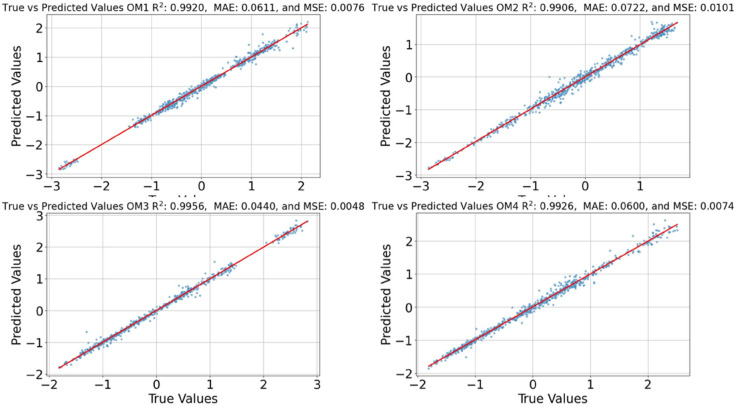
Predicted vs actual values of the first 4 modes.

Final results of the optimised models are shown in Table 3. After analysing these results, SVM emerged as the best-performing algorithm, achieving an ${\text{R}}^{2}$ of 0.95 on the test set, with an MAE of 0.122 and an MSE of 0.042. Fig 12 displays the prediction plot for the different modes.

**Table 3:** ${\text{R}}^{2}$, MSE, and MAE of the optimised algorithms.

Model	${\text{R}}^{2}$	MSE	MAE
**RF**	0.930	0.058	0.156
**XGB**	0.929	0.059	0.154
**SVM**	0.950	0.042	0.122
**GB**	0.939	0.050	0.137

### Validation

**Fig 13:**
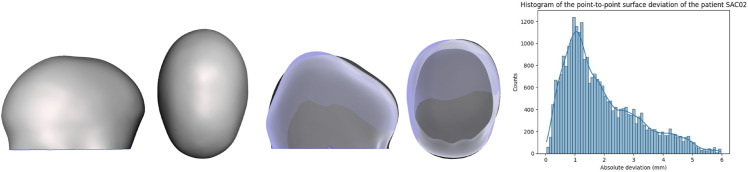
Preoperative 3D Photograph (left), follow-up vs prediction (middle) and distribution of the surface error (right) of a patient with an average error of 1.88 mm.

**Fig 14:**
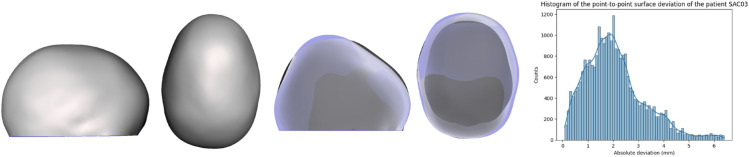
Preoperative 3D Photograph (left), follow-up vs prediction (middle) and distribution of the surface error (right) of a patient with an average error of 2.07 mm.

**Fig 15:**
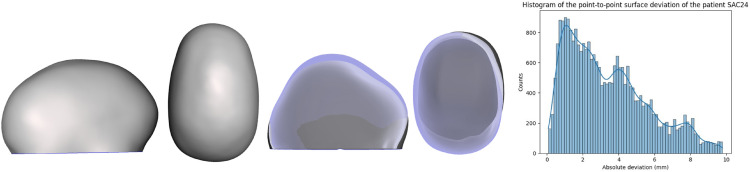
Preoperative 3D Photograph (left), follow-up vs prediction (middle) and distribution of the surface error (right) of a patient with an average error of 3.39 mm.

**Fig 16:**
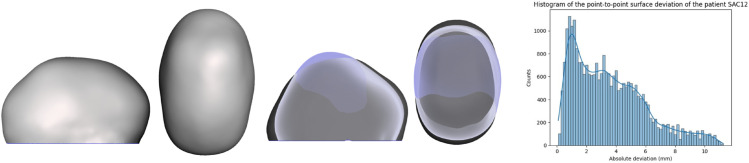
Preoperative 3D Photograph (left), follow-up vs prediction (middle) and distribution of the surface error (right) of a patient with an average error of 3.62 mm.

Although the algorithms demonstrated accurate results in predicting synthetic data generated by FEM, their performance declined when compared to the actual surgical output. The average metrics of the validation set are: ${\text{R}}^{2}$ of -3.58, MSE of 1.41 and MAE of 0.88. Several factors not included in the FEM, such as growth, may have contributed to the observed differences. Figs 13 and 14 show the validation patients predicted wit the lowest average surface error. Figs 15 and 16 show the validation patients predicted with the greatest average surface error.

**Fig 17:**
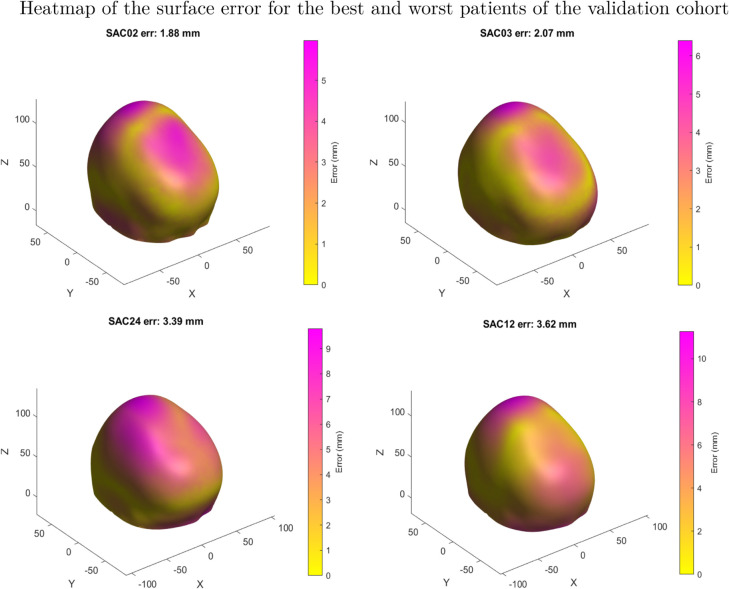
Heatmap showing the surface error distribution of the aforementioned patients.

Finally the error distribution on the surface can be observed on the heatmap displayed on Fig 17.

## Discussion

This paper presents a novel ML-FEM approach to enhance pre-operative planning and outcome predictability for patients undergoing SAC. By leveraging simplified skull models derived from population means, this method facilitates the future use of radiation-free 3D photographs instead of CT scans for preoperative planning. Currently, only 25% of patients at GOSH receive preoperative imaging to mitigate the risk of tumours later in life [23].

The feasibility of FEM for predicting surgical outcomes in SAC has been established, validated, and optimized by the research group [9–11,21]. While traditional numerical models depend on CT imaging data, this study introduces a novel approach that incorporates 3D photographs, thereby eliminating the need for radiation and anaesthesia in young patients. The manual set-up for FEM requires approximately one hour for NURBS creation, and up to 5 hours of simulation to achieve follow-up results for a single surgical configuration.

Surrogate models trained on FEM data have demonstrated success in other medical fields, such as cardiology [12], showcasing their potential for real-time predictions. The developed ML model achieves an ${\text{R}}^{2}$ accuracy of 0.95, with MSE and MAE values below 0.13. Despite being trained on synthetic data, it performs real-time predictions with an error rate of 2.70 ± 0.58 mm. This accuracy is comparable to that reported by [21], where 80% of the points had an error below 2 mm, while also speeding the process and eliminating the need for radiation.

The errors on the validation cohort ranged from 1.88 mm (Fig 13 to 3.62 mm (Fig 16), with an average of 2.70±0.58 mm. A correlation analysis was performed to investigate potential factors influencing this variability. Age showed a moderate positive correlation with error (r = 0.58), suggesting that younger patients tended to have slightly lower errors. The CI was negatively correlated with error (r = –0.31), indicating that patients with more severe craniosynostosis (lower CI) exhibited higher errors. Regarding surgical configurations, the use of stiffer springs was positively correlated with error (r = 0.61 for anterior and r = 0.46 for posterior springs). These trends may reflect the increased biomechanical complexity in older patients, in more severe craniosynostosis, and under higher spring forces. However, given the limited sample size, these correlations should be interpreted with caution, and further validation on larger cohorts is required to confirm their significance.

Several assumptions contribute to the model’s final errors and the observed performance drop on the validation set. First, the training dataset consisted of a relatively small set of head CT images, while validation was conducted using 3D photographs, resulting in a domain shift between the training and validation modalities. The validation set was projected into the PCA space derived from CT data which also introduces registration related errors. Furthermore, when feeding the validation data, the model does not account for patient growth between surgery and follow-up, necessitating re-scaling under the assumption of unchanged volume which also contributes to the final error. In addition, the use of standardized material properties across the cohort, the absence of suture tearing considerations, and the uniform application of obtained *t*_*skull*_ and *t*_*skin*_ values to all patients neglect the individual variabilities. The aforementioned factors are likely responsible of the performance drop and highlight areas for future model refinement.

Dimensionality reduction techniques should also be further analysed. PCA is a linear dimensionality reduction technique, when not all modes are maintained some explained variance is lost which also contributes to the model error. However, keeping high amount of modes to maintain all the explained variance and hence increasing the dimensionality of the dataset compromises the so called curse of dimensionality introduced by [24].

Future work aims to address these assumptions and thereby reduce errors. Models capable of inferring skull structures from 3D photographs [25] will enable the application of the already validated numerical models to more sophisticated synthetic skulls, moving beyond uniform offsets or population means. Recent advances in generative AI models could, in principle, provide alternative means to simulate surgical outcomes. However, such models typically require much larger datasets and substantial computational resources, and our available dataset (single surgery per patient with specific configurations) was insufficient to realistically train them. Therefore, we focused on a conventional ML approach built upon FEM-generated data, which allowed accurate predictions even with limited patient numbers.

The synthetic 80 scenarios generated during the DoE stage for each patient introduced variability into the model, allowing it to learn different possible surgical configurations and their effects on the final outcome. Training on real patient data might have improved validation accuracy by reducing modelling errors. However, the limited number of patients and the similarity of surgical configurations would have resulted in a smaller dataset with lower variability.

Although the initial training pipeline is time-consuming and computationally demanding, as it requires FEM simulations across different surgical scenarios, the inference stage is efficient. For a new patient, only registration with the template, projection into the PCA space, and specification of the surgical configuration are needed. The trained model then generates the predicted postoperative shape within seconds.

Currently, the model is capable of real-time predictions of the follow-up results only relying in 3D photographs, in some iterations it will offer clinicians valuable guidance during SAC pre-operative planning. The use of surrogate models has significantly reduced computational time while maintaining predictive accuracy, enabling surgeons to make informed decisions efficiently and to choose the best surgical configuration for each of the patients. This advancement not only enhances preoperative planning but also improves patient and family experience by providing a reliable visual representation of expected surgical outcomes.

Nevertheless, it is essential to acknowledge the study’s limitations. The model was trained and tested on FEM output, disregarding factors such as scalp growth and relying on population-averaged values instead of personalized parameters. These simplifications have led to discrepancies between the synthetic data used for ML model training and actual surgical outcomes. Despite this, the ML model can predict surgical outcomes with an error of 2.70±0.58 mm, delivering results comparable to FEM simulations within seconds.

## Conclusion

In conclusion, despite its current limitations, this study underscores the potential of ML-based models in predicting surgical outcomes in the craniofacial context. Future iterations incorporating real patient data and more realistic simulations are expected to enhance accuracy and reliability, further establishing ML as a valuable tool in preoperative surgical planning for SAC.
